# The Development, Description and Appraisal of an Emergent Multimethod Research Design to Study Workforce Changes in Integrated Care Interventions

**DOI:** 10.5334/ijic.2510

**Published:** 2017-03-08

**Authors:** Loraine Busetto, Katrien Luijkx, Stefano Calciolari, Laura G. González-Ortiz, Hubertus J.M. Vrijhoef

**Affiliations:** 1 Tranzo Scientific Center for Care and Welfare, Tilburg University, Warandelaan 2, 5037 AB Tilburg, NL; 2 Institute of Economics (IdEP), Università della Svizzera Italiana, Via G. Buffi 13, CH-6904 Lugano, CH; 3 Panaxea B.V., Amsterdam, NL; 4 Department of Patient and Care, Maastricht University Medical Center, NL; 5 Department of Family Medicine, Vrije Universiteit Brussels, BE

**Keywords:** multimethod research, emergent design, qualitative research, integrated care, workforce changes

## Abstract

**Introduction:**

In this paper, we provide a detailed and explicit description of the processes and decisions underlying and shaping the emergent multimethod research design of our study on workforce changes in integrated chronic care.

**Theory and methods:**

The study was originally planned as mixed method research consisting of a preliminary literature review and quantitative check of these findings via a Delphi panel. However, when the findings of the literature review were not appropriate for quantitative confirmation, we chose to continue our qualitative exploration of the topic via qualitative questionnaires and secondary analysis of two best practice case reports.

**Results:**

The resulting research design is schematically described as an emergent and interactive multimethod design with multiphase combination timing. In doing so, we provide other researchers with a set of theory- and experience-based options to develop their own multimethod research and provide an example for more detailed and structured reporting of emergent designs.

**Conclusion and discussion:**

We argue that the terminology developed for the description of mixed methods designs should also be used for multimethod designs such as the one presented here.

## Introduction

### Workforce changes in integrated care interventions

The demand for health care is on the rise and changing from acute, short-term care to chronic, long-term care [1,2]. This is mainly due to population ageing, increased prevalence of lifestyle factors conducive to chronic disease and a change in the definition of illness to include also those at risk of disease [1,2,3]. Integrated care has been suggested as a means to approach these challenges and its delivery is a priority in many countries’ efforts to improve health outcomes for people at risk of or with chronic illness [3,4,5]. Integrated care is an approach that seeks to improve the quality of care for patients with long-term illness by ensuring that services are well-coordinated around their needs [6]. Generally, integrated care concerns complex interventions which include changes to patient-provider relationships, care process designs, communication infrastructures and to how health professionals deliver care [7]. The World Health Organization defines integrated care as ‘the management and delivery of health services such that people receive a continuum of health promotion, health protection and disease prevention services, as well as diagnosis, treatment, long-term care, rehabilitation, and palliative care services through the different levels and sites of care within the health system and according to their needs’ [8]. In line with previous research, we operationally defined integrated care as interventions targeting at least two components of the Chronic Care Model [9,10,11].

Within the scope of Project INTEGRATE on “Benchmarking Integrated Care for Better Management of Chronic and Age-related Conditions in Europe”, we studied what constitutes good quality integrated care provision. As part of this project, five so-called “cross-cutting” issues were studied, that were expected to play an important role in the delivery of integrated care. These included care process design, financial flows, patient involvement and information technology (IT) management and workforce changes. We were the work package leader of the study on workforce changes. Specifically, our objective was to investigate (1) the workforce changes implemented as part of integrated care interventions for people with chronic conditions, (2) the barriers and facilitators to their implementation, as well as (3) their outcomes [12]. In this paper, we only focus on the development of the research design to study the above aspects. As the results are reported in detail elsewhere [13], we only provide a short summary in the current manuscript.

Given health professionals’ involvement in all aspects of integrated care, it is assumed that changes to the health workforce affect the implementation of integrated care profoundly. By “health workforce” we mean “the different kinds of clinical and non-clinical staff responsible for public and individual health intervention” [14]. Workforce changes are those changes experienced by the health workforce [12]. Previous research has investigated health workforce planning [15,16], present and future health workforce needs [17], trends for specific sectors or groups of health professionals [18,19], and specific types of changes for the health workforce such as skill mix or team work [20,21]. There is, however, a lack of studies conducted specifically on integrated care interventions for chronic diseases. This is problematic because chronic care focusses on long-term management of illness and therefore differs considerably from acute care with its focus on episodic treatment of illness. Moreover, when workforce changes are implemented as part of multi-component interventions, they are implemented in combination with changes targeting the other areas of integrated care delivery described above. Studying workforce changes as part of these complex interventions requires the use of study designs that are able to capture this complexity.

### Emergent multimethod research designs

Health services research is increasingly concerned with the study of complex interventions that include multiple components, target multiple levels, involve multiple actors and contribute to multiple outcomes [22]. By combining more than one method of research within the same study, multimethod research is considered especially appropriate for the study of complex interventions [23,24,25,26]. Multimethod research designs may be the outcome of prescriptive planning (i.e. enacted) or an unfolding, evolutionary, pragmatic research process (i.e. emergent) [24]. The latter, more flexible approach, refers to designs whose detailed frameworks emerge during the study, depending on the data and the researchers’ interpretation thereof [27]. A defining characteristic of emergent designs is that they are flexible by allowing for interaction between different strands of data at different points of time during the research [28]. For example, one can use the preliminary findings from one data collection as a basis for the next data collection or as a framework for its analysis.

The choice for an emergent design can be a conscious one at the outset of the research to let the data guide the next steps of the research [29], but it is also possible that the decision is necessitated at some point during the project at which the research is moving in a different way than originally anticipated [27,30,31]. In both cases it would be useful to learn which choices were made by researchers in particular situations and why, or which difficulties were encountered and how these were overcome. Unfortunately, detailed accounts of emergent research processes in scientific papers are rare. Instead, most readers have to contend with the simple notion that an emergent design was used and the presentation of the route that “worked” [30]. As a remedy, scholars have argued that there should be more focus on the actual research process, in particular on the actual integration of multiple methods, rather than the “reconstructed logic” of published designs and methods [32]. In order to better understand how and to what end multiple methods were integrated, Maxwell et al. have urged researchers to write about their experiences with integrated approaches and methods [32].

### Research objectives

The first aim of this paper is therefore to provide a detailed and explicit description of the process and decisions underlying and shaping the emergent multimethod research design of our study of workforce changes in integrated care for people with chronic conditions. However, even when detailed descriptions are available, these tend to concern retrospective accounts of one specific research project as well as the problems encountered and solutions found for this specific context only [27,29,30,33]. While this is certainly useful, it does not necessarily help other researchers challenged with similar problems in other settings. It would be more useful to characterise the research design as a specific type of multimethod design and thereby provide researchers with a more general set of theory- and experience-based options that can be followed throughout an emergent research process and labelled as such in its description [32]. Leeman et al. have added that graphic presentations of the approaches used are especially useful for multimethod research [22]. The second aim of this study is therefore to schematically display the interdependencies of the different data strands included in the design and thereby identify the type of multimethod design that was used.

In the following, we describe our initial plan for data collection and analysis. This is followed by a detailed description of why and how this plan was changed, including a reflective commentary [34]. Next, a graphic display of the research design is presented. We also present a short summary of the results before ending with a discussion of the limitations, strengths and implications for further research.

## Initial data collection and analysis

**Figure 1:**
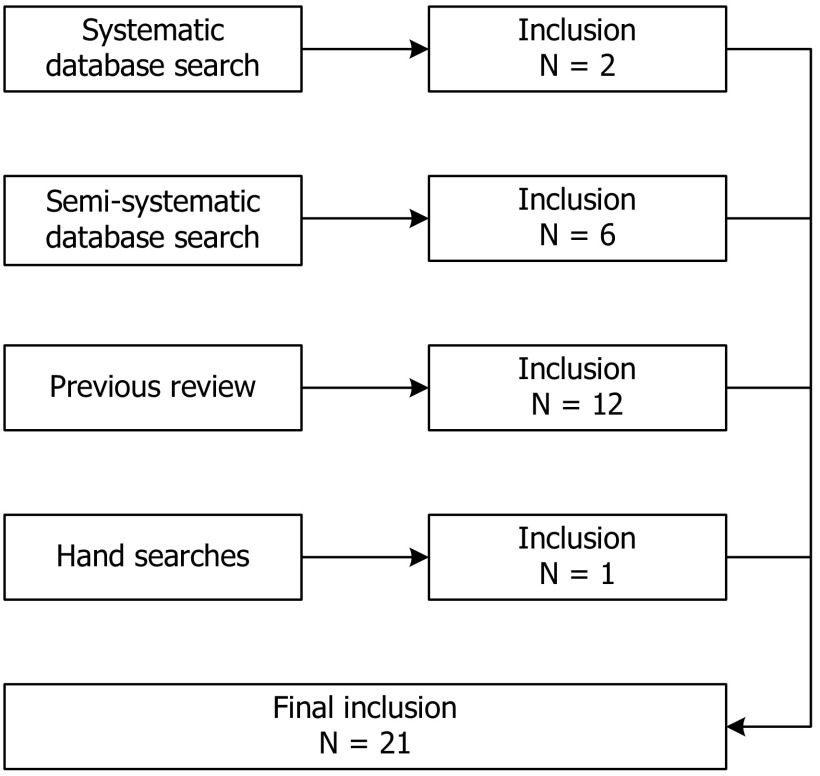
Flowchart of the literature review selection process.

Initially, we planned to conduct a systematic literature review followed by a Delphi panel. The purpose of the combination of the two methods was triangulation for the convergence and corroboration of findings [24]. First, the data collection for the literature review was to be conducted, followed by the data analysis and interpretation of results. Based on the literature review, we envisaged to find a list of workforce changes, several barriers and facilitators, and positive and/or negative outcomes of these workforce changes. We planned to use these findings as the basis for the data collection of the Delphi panel by using them as input for the items to be confirmed, rated or commented upon by the experts within the scope of the Delphi panel. The first step of the research was to design a literature review strategy and to conduct the database searches. We employed the following four-step approach for the literature search: (a) systematic database search; (b) semi-systematic database search; (c) secondary analysis of a previous literature review; and (d) unsystematic hand search. Three groups of search terms were used in the search, relating to chronic conditions, intervention type, and workforce. The complete list of search terms is reported elsewhere [12]. All articles were subjected to title, abstract and full text scans, performed individually by three researchers (LB, SC, LG) and then discussed together until consensus was reached. Figure 1 depicts the selection process of the four stages of the literature review. The final selection consisted of 21 studies. Initially, we only analysed the workforce changes that were found in the articles included in the literature review. A list of common workforce changes was compiled from the literature by one researcher (LB) and checked independently by two researchers (SC, LG). The list was discussed and adapted until all researchers agreed that all workforce changes from the included studies were covered and there were no redundancies in the list.

## Revision of the research plan

At this point of the research, we considered it necessary to re-evaluate the original research plan. As Figure 1 shows, only two studies were identified from the initial, systematic literature search. Adding additional steps made it possible to retrieve more articles and, eventually, to identify a set of workforce changes. But given the fact that most articles were retrieved through the unsystematic searches, it was not clear to what extent these findings could be generalised and whether they were complete. It was therefore decided that a quantitative validation of this list via a Delphi panel would not be a productive approach. Instead, we chose to continue our qualitative exploration of the topic, specifically by conducting a qualitative expert questionnaire and secondary analysis of two case reports of best practice integrated care implementation. The purpose of the combination of multiple methods changed from triangulation for convergence and corroboration to complementarity for elaboration and enhancement of findings [24].

Between January and April 2015, we administered a qualitative exploratory questionnaire to experts in the fields of integrated care, chronic care, and health human resource management. We included experts with academic or policy backgrounds as well as ‘field experts’ (i.e. health professionals or managers of organisations involved in the provision of integrated care). Experts were identified using the snowball method and had to be proficient in English or in one of the three languages in which translations of the English questionnaire were available (i.e. Dutch, Italian and Spanish). In total, the questionnaire was sent to 91 experts, of which 25 completed and returned the questionnaire, resulting in an overall response rate of 28%. Experts were asked to indicate which of the workforce changes from the literature review they recognised from their own experience and which were missing. Moreover, they were requested to describe an integrated care intervention, the workforce changes included in this integrated care intervention, the barriers and facilitators to the implementation of the workforce changes, and the outcomes of the workforce changes. Apart from the information from the literature review and expert questionnaires, we wanted to know whether the same or possibly more workforce changes, barriers, facilitators and outcomes could be found when looking in detail at several best practices of integrated care implementation. We had access to two detailed case reports that included information on a specific integrated care intervention including workforce changes and the barriers and facilitators to their implementation. The case reports were written as part of an earlier phase of Project INTEGRATE. The data extraction of these reports was sent to the authors for their confirmation and feedback. All changes and comments provided were taken up in the analysis of the case reports. One of the case reports has been published elsewhere [49].

We also decided that we would not complete the analysis for one data source and then move on to the next data source, and so on. Instead, we decided to perform the data analysis for each research objective separately, i.e. first for the workforce changes, second for the barriers and facilitators, and third for the outcomes. This choice was based on the decision to use the CMO Model (context + mechanisms = outcomes) as umbrella framework for our research. The CMO Model states that interventions only have successful outcomes when they introduce the appropriate mechanisms in the appropriate context. By focusing on the intervention (workforce changes), the implementation process (barriers and facilitators) as well as the outcomes, the CMO Model is especially appropriate for investigating complex interventions such as integrated care [35,36,37]. Following from this, it was necessary to study the elements of the CMO Model in coherence with each other. Mechanisms were operationalised as interventions, context as barriers and facilitators to the implementation of the intervention, and outcomes as effects triggered by mechanisms and context [13]. Workforce changes were seen as part of the integrated care intervention and therefore as part of the mechanisms.

### Workforce changes

The data analysis of the workforce changes from the expert questionnaires was performed by four researchers (LB, KL, SC, LG). We used the list obtained from the literature review as initial coding list for the coding of the questionnaire workforce changes. The coding list could be expanded and adapted when necessary. For the case report workforce changes, a secondary analysis was performed in which the workforce changes described in the case reports were mapped against the coding list identified from the literature review described above. When the analyses of the workforce changes from the literature review, expert questionnaires and case reports were finalised, we compared the workforce changes that were reported in both case reports or among those mentioned by most studies and experts as evidenced by their belonging to the three highest percentages per data source. This resulted in a list of seven workforce changes.

### Barriers and facilitators

After the analysis and interpretation of the workforce changes was completed, we turned to the barriers and facilitators. We started by analysing the data on the expert questionnaire barriers and facilitators because these were found to be richer in volume and content than the data on barriers and facilitators collected through the literature review or available in the case reports. An open coding approach was used which meant that two researchers (LB, KL) independently created a coding list and then compared and consolidated the coding lists together. To build on the coding process conducted for the expert questionnaire barriers and facilitators, the data from the literature were coded based on the coding lists resulting from the expert questionnaire. When the data did not fit the categories of the coding lists, the list was adapted accordingly. The case report barriers and facilitators were coded using the adapted coding list from the expert questionnaire and literature review. Finally, we compared the barriers and facilitators that were reported in both case reports or among those mentioned by most studies and experts as evidenced by their belonging to the three highest percentages per data source. This resulted in six categories of facilitators and six categories of barriers to the implementation of workforce changes.

### Outcomes

After the data collection, analysis and interpretation for the barriers and facilitators was completed, we turned to the outcomes of the workforce changes. We started by analysing the data from the expert questionnaires because the questionnaires already included a list of outcome categories which could be used as a basis for the coding list. Four researchers were involved in the data extraction and analysis (LB, KL, SC, LG). For the literature review outcomes, the data were coded based on the coding list resulting from the expert questionnaire. When the results from the literature review did not fit into the categories of this coding list, the list was adapted accordingly. The case report outcomes were coded using the adapted coding list from the expert questionnaire and literature review. As the case reports did not report outcomes of the interventions, this data strand was not included. Finally, we compared the outcomes that were among those mentioned by most experts or studies, as evidenced by their belonging to the three highest percentages per data source. Positive and negative outcomes were found for five different categories.

## Transcending the particular

**Figure 2:**
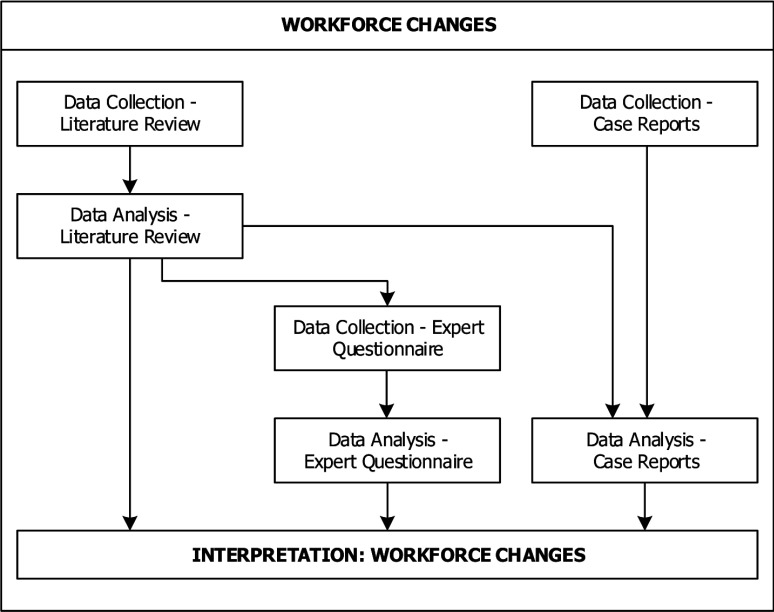
Adapted research design: workforce changes.

**Figure 3:**
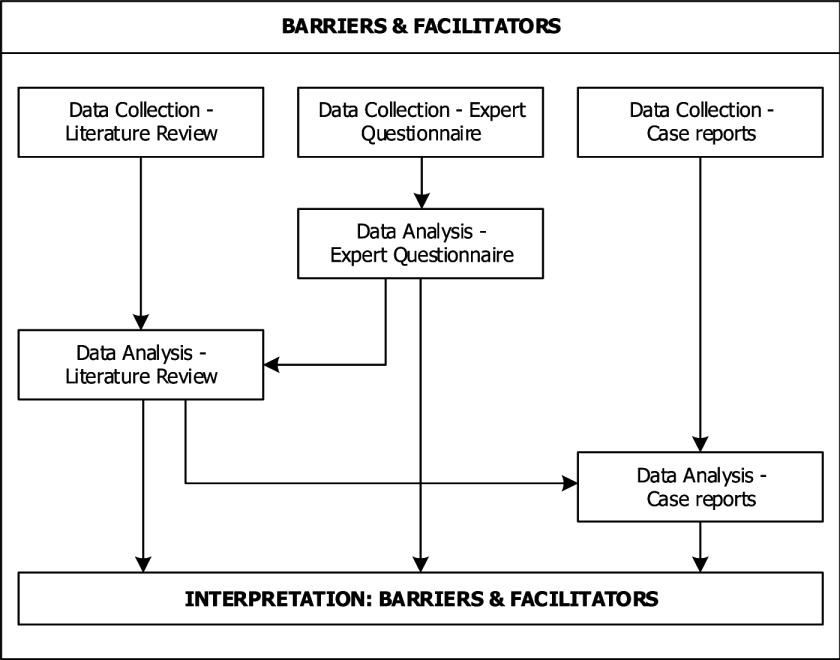
Adapted research design: barriers & facilitators.

**Figure 4:**
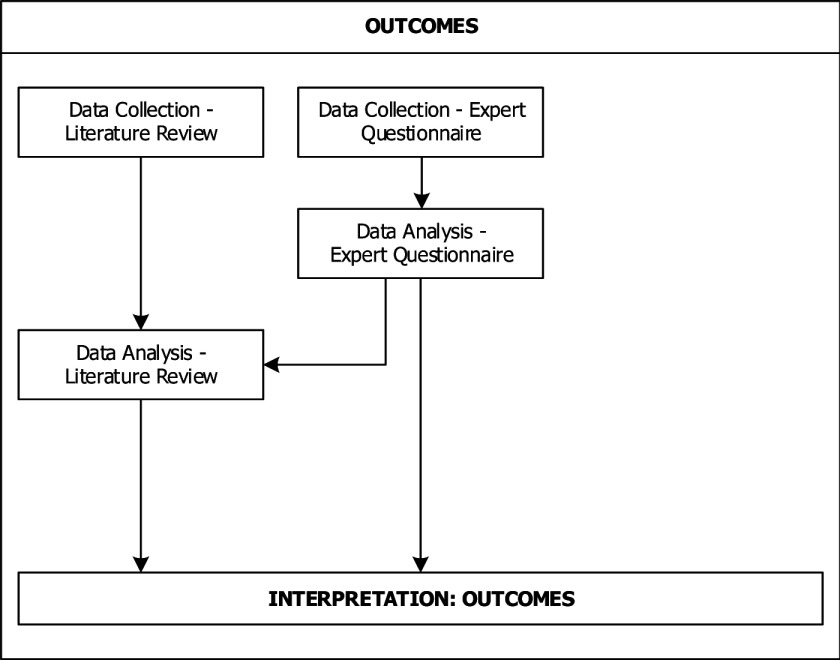
Adapted research design: outcomes.

After the finalisation of these steps, we had collected, analysed and interpreted data from three different sources. In order to turn our own experience into a useful learning opportunity for ourselves and others, we decided to describe the research design that resulted from this process in a schematic way. In the following we present a graphic display of the research design including the sequencing and (in-)dependencies of the data strands as regards the collection, analysis and interpretation of data in the adapted research design. Figures 2, 3, 4 show how our emergent multimethod research design combined different data strands in an interactive manner with multiphase combination timing. By *data strands* we refer to the processes of data collection, analysis and interpretation. *Interactive* designs are characterised by direct interaction between the different data strands, for example when the results of one data collection are used as basis for another data collection. *Multiphase combination timing* combines concurrent timing (when the data strands are carried out during a single phase of the study) and sequential timing (when the data strands are carried out in two distinct phases, and one strand is carried out after the other) [28]. Figure 2 concerns the workforce changes, Figure 3 the barriers and facilitators and Figure 4 the outcomes.

Figure 2 describes the data collection and analysis to identify workforce changes by means of a literature review, expert questionnaires and case reports. Here, the data collection for the expert questionnaire was dependent on and sequential to the data collection and analysis of the literature review, because the list of workforce changes from the literature review was incorporated into the data collection for the expert questionnaire by asking experts to confirm and/or complete the list of workforce changes. The data analysis of the workforce changes for the expert questionnaire and the secondary analysis of the case reports were both dependent on and sequential to the data collection and analysis of the literature review, because the results from the literature review were also used for data analysis of the expert questionnaires by serving as coding list to analyse and categorise the experts’ descriptions of the workforce changes in the integrated care interventions. The same holds true for the secondary analysis of the case reports, where the list from the literature review also served as a coding list for the workforce changes described in the case reports. However, the data collection for the case reports was carried out concurrently and independently of the data collection and analysis for the literature review. The results from all three data strands were combined in the end at the stage of interpretation.

Figure 3 describes the data collection and analysis for the barriers and facilitators to the implementation of workforce changes. Here, the data collection for the literature review, expert questionnaires and case reports were carried out concurrently and independently of each other. It might seem contradictory that Figure 2 seems to show the data collection for the expert questionnaire to have taken place *after* the data collection for the case reports, while Figure 3 seems to show all data collections to have taken place *at the same time*. However, this would be a misunderstanding of the difference between the actual *timing* of a specific activity (i.e. the day/month/year it was carried out) and the *sequencing* of a specific activity (i.e. whether it was carried out independently or dependently of a previous activity).

To use a specific example, the data collection for the literature review was carried out in August/September 2014 and the data collection for the expert questionnaire between January and April 2015. In the case of the workforce changes, the actual *timing* is relevant because the data collection for the expert questionnaire was dependent on the data collection and analysis of the literature review, which means that the data collection for the expert questionnaire had to take place *after* the data collection and analysis of the literature review. However, even though the timing is relevant, it should be noted that Figure 2 (as do Figures 3 and 4) shows the *sequencing* of the activities, i.e. the fact that the data collection for the expert questionnaire was sequential to the data collection and analysis for the literature review. As regards the barriers and facilitators, the *timing* of the data collection for the literature and expert questionnaire is not relevant because the two activities were carried out independently of each other. This means that it does not matter that the actual *timing* of the data collection was approximately half a year apart, because the data collection activities were carried out concurrently.

Figure 3 describes the data collection and analysis for the outcomes of workforce changes. Again, the data collection for the literature review, expert questionnaires and case reports were carried out concurrently and independently of each other. As a next step, the data analysis for the expert questionnaires was carried out by using a coding list based on the specific answer categories used in the questionnaire. This coding list was used for and adapted based on the coding of outcomes from the literature review. As in the two previous cases, the results from the two data strands were combined at the final stage of interpretation.

## Summary of results

As mentioned earlier, the results of the studies that are based on the above research design are reported in detail elsewhere [12,13]. In short, we found seven workforce changes that were implemented as part of integrated chronic care interventions, namely (1) nurse involvement in the delivery of care; (2) multidisciplinary staff including health professionals from different disciplines; (3) multidisciplinary protocols/pathways involving tasks for health professionals from different disciplines; (4) provider training such as on-the-job training or educational seminars or materials for health professionals; (5) involvement of a case manager/care coordinator role in the delivery of care; (6) regular team meetings to discuss a patient’s treatment; and (7) the creation of a new position, role or function specifically to deliver integrated chronic care. Moreover, most barriers related to problematic delivery structures, health professionals’ skills and enthusiasm, IT, funding, culture and cooperation and communication. Most facilitators related to health professionals’ motivation and enthusiasm, good delivery structures, communication and cooperation, IT, patients, leadership and senior management. Finally, we found mostly positive outcomes, in particular for quality of care (including clinical patient outcomes and process measures), patient satisfaction and staff satisfaction.

## Discussion and appraisal

This paper described the development of an emergent and interactive multimethod design with multiphase combination timing to investigate the workforce changes implemented as part of integrated care interventions for people with chronic conditions, the barriers and facilitators to their implementation, as well as their outcomes. The original research plan foresaw an initial investigation of the workforce changes, their barriers and facilitators, and their outcomes as described in the international scientific literature. We planned to confirm these findings quantitatively via a Delphi panel among an international group of experts in the field of integrated chronic care and health human resource management. However, when the systematic literature review yielded only a very small number of studies, we decided to continue the qualitative exploration of the topic instead of aiming for a quantitative confirmation of first results, whose generalisability and completeness were in doubt. Consequently, the Delphi study was replaced by a qualitative expert questionnaire and two qualitative case reports of best practice examples of integrated care implementation.

Glavare, Löfgren and Schult [38] describe an emergent design used in a qualitative study on the experience of unemployed long-term pain sufferers. As they only used one method of data collection (interviews), the emergent nature of their design lay in the use of preliminary topics resulting from the coding of early interviews for the adaptation of the topic list used in later interviews. Other studies describe similar uses of emergent designs within the framework of one method of data collection [39,40,41]. In their study on the management of spinal cord injury neuropathic pain, Norrbrink & Löfgren [42] used an emergent design that started with focus groups among patients. When the preliminary findings from these focus groups indicated the need to involve the health care perspective, individual interviews were conducted with physicians. The interview guide for these interviews was based on the first results of the focus group analysis, which were also used for the analysis of the physician interviews. This process is similar to the interaction between data collection and analysis that we described for the analysis of workforce changes in the literature review that was used in the data collection for the expert questionnaires as well as for the analysis of workforce changes from the expert questionnaires and case studies. Apart from these examples, little has been published in scientific journals on the topic of emergent designs. As mentioned earlier, those studies that do employ emergent designs tend to offer only very limited descriptions or else detailed descriptions that only apply to their own specific circumstances.

While we deemed a change of plans necessary in our particular situation, this was not without drawbacks, the main one being that we were not able to perform a quantitative analysis of our qualitative findings. Ideally, we would have wanted to perform more qualitative research first, and then return to the original plan of quantitative conformation of the qualitative results. However, conducting qualitative research is time-consuming and adding not one but two additional qualitative elements to our design meant that this was really an either-or-decision instead of an add-on to the original plan, at least within the scope of this particular project. Given this limitation, we recommend further quantitative exploration of our research findings for future research, for which we believe our qualitative research provides a much stronger basis than the original research design would have allowed. A second disadvantage of the adapted research design is related to the decision to focus on the three research objectives (workforce changes, barriers and facilitators, and outcomes) separately. Given this specific set-up, we were able to collect, analyse and interpret data for each research objective separately, but not in combination. This means that while the research design allowed us to gain insights on the different types of workforce changes, the barriers and facilitators, and outcomes separately, it does not allow us to find out which types of workforce changes are generally encountered by which barriers and facilitators or related to which positive or negative outcomes, which would be valuable information for practitioners and policy-makers in the field of integrated care. However, this type of detailed information might not be a realistic goal for an exploratory study as described here. Instead, we are confident that our findings will provide detailed and evidence-based input for more detailed quantitative and qualitative investigations of the relationships between the workforce changes, the barriers and facilitators to their implementation, as well as the outcomes achieved. This could be done in combination with further quantitative exploration of our findings. We would also recommend increased emphasis on identifying which key actors are involved in workforce changes to enable the inclusion of all relevant stakeholders in future research.

Despite these drawbacks, there were also several important advantages resulting from the change of research design. One of the main advantages is the fact that it allowed us to make use of the data gathered via the literature review, which contributes to the study’s information-richness [43]. As explained earlier, when the initial systematic search only led to the inclusion of two studies, we added unsystematic steps which resulted in the inclusion of 19 additional studies which held rich and relevant results for our research questions. However, since these results were for the largest part not based on a systematic search, it was impossible to know to what extent the findings are complete or whether they only paint a partial picture. Confirming this potentially partial picture quantitatively did not seem like worthwhile option. Of course, this did not mean that the review findings said nothing; it just meant that we did not know whether they said *enough*. Switching to a different multimethod approach allowed us to still make use of the information we had from the literature review by using it as input for further data collection, as coding lists for data analysis and point of reference for the interpretation of overall results. As Daly et al. [44] point out, literature reviews are generally the starting point for more complex and more advanced studies.

This relates to a second strength of the new study design: its flexibility. Switching from a fixed design to a flexible one allowed us to combine different data strands at different points of time during data collection and analysis. As Archibald et al. pointed out in their review of current mixed methods practices in qualitative research, 98% of the studies they reviewed mixed data at the interpretation stage only [45]. In our study, however, we combined different methods during all stages of the research, which made it possible to build upon and triangulate insights gained from earlier data strands and further explore concepts that became apparent during early data collection and analysis phases. The combination of different methods remedies their respective limitations while at the same time enhancing their respective advantages and thereby contributes to the credibility of the study [24,34,46]. Additionally, this triangulation improved the confirmability of our study by limiting investigator bias inherent to qualitative research [34,46]. Furthermore, our study design combines in depth data from the case reports with a broad scope of data from the literature review and expert questionnaire. It also includes very local data from the case reports with regional and national data from the literature review, while the expert questionnaires provided local, regional and national data. Including data from different environments contributes to the transferability of study findings to other settings [34].

The most compelling argument in favour of the adapted research design presented here is the fact that it allowed us to still achieve our research objectives. We were able to identify seven types of workforce changes implemented as part of integrated care interventions for people with chronic conditions, six categories of barriers and facilitators to the implementation of workforce changes, as well as five categories of negative and positive outcomes. We are convinced that this would not have been possible by staying with the original research plan. Furthermore, we were able to retrospectively describe the multimethod design that emerged over the course of the research in a schematic way and label it as an emergent and interactive research design with multiphase combination timing. In doing so, we transcend the particulars of our own study and make our strategies and options accessible for future projects by other researchers. To use Shenton et al.’s [34] words, we created a “prototype model” of a research design which allows for theoretical or actual repetition of our work and which thereby contributes to the dependability of our study.

The terminology used to label the different parts and processes of the emergent design in this paper was originally developed for, and is currently used to describe, mixed methods research, that is, the combination of quantitative and qualitative methods [28,47,48]. However, we recommend that this terminology – and the ideas and concepts it describes – should be used for any design that combines different data strands, whether they be quantitative, qualitative or a mix of both. The common denominator is that one has to be clear and specific about the choices that were made in the combination of these data strands, at which points they took place and what consequences they had for the overall research objective. Because, as Fossey et al. [43] explain, this “enables the reader to understand the intentions of the study and evaluate the congruence (fit) between these intentions and subsequent choices”. By providing detailed insights into the processes and decisions regarding the collection, analyses and interpretation of data, including both the road that did and did not work, this paper contributes to increased transparency and transferability of methods to be used in complex study designs. By outlining and labelling the options available in the course of an emergent design, we expect our account to be of assistance to researchers planning or presently conducting multimethod research. We also hope that our account can act as an example for a more detailed and structured reporting of emergent research designs.
